# Muscle Energy Technique in the Rehabilitative Treatment for Acute and Chronic Non-Specific Neck Pain: A Systematic Review

**DOI:** 10.3390/healthcare9060746

**Published:** 2021-06-17

**Authors:** Silvia Sbardella, Chiara La Russa, Andrea Bernetti, Massimiliano Mangone, Andrea Guarnera, Letizia Pezzi, Marco Paoloni, Francesco Agostini, Valter Santilli, Raoul Saggini, Teresa Paolucci

**Affiliations:** 1 Department of Anatomy, Histology, Forensic Medicine and Orthopedics, Sapienza University, 00185 Rome, Italy; silvia.sbardella@uniroma1.it (S.S.); chiara.larussa@uniroma1.it (C.L.R.); andrea.bernetti@uniroma1.it (A.B.); massimiliano.mangone@uniroma1.it (M.M.); andrea.guarnera@uniroma1.it (A.G.); marco.paoloni@uniroma1.it (M.P.); valtersantilli@uniroma.it (V.S.); 2 Department of Medical, Oral and Biotechnological Sciences (DSMOB), G. D’Annunzio University of Chieti-Pescara, 66100 Chieti, Italy; letizia.pezzi@unich.it (L.P.); raoul.saggini@unich.it (R.S.); teresapaolucci@hotmail.com (T.P.)

**Keywords:** neck pain, management, rehabilitation, muscle energy technique, upper trapezius trigger points, systematic review

## Abstract

Background: Non-specific neck pain (NNP) affects 30–50% of the general population, and it often leads to severe disability. Several manual therapy techniques are available to reduce pain and disability and to improve cervical range of motion and functional activities. Muscle Energy Technique (MET) showed more evidence for treating such a disorder. The aim of this current scientific literature analysis was to compare the clinical effects of MET with the other manual or rehabilitative treatments for non-specific acute and chronic neck pain. Methods: The literature search was conducted using the following databases: PubMed, Medline, PEDro, Cochrane Database, and Google Scholar from 2010 to January 2020. Clinical trials about MET were included. The quality of the trials was assessed according to the PEDro scale. Results: Twenty-one papers according to inclusion and exclusion criteria were selected: 15 studies about non-specific acute neck pain and 6 studies about non-specific chronic neck pain. Conclusions: This analysis suggests that the MET approach has a good clinical effect on reducing neck pain in patients with acute neck pain and improves cervical range of motion in patients with chronic neck pain, and is better if combined with a traditional rehabilitative approach. This review’s findings should be considered with caution for physiotherapy practice because of the studies’ methodologic limitations. On the basis of the current available and limited evidence, clinicians could combine MET with traditional physiotherapy and other manual techniques when treating people with non-specific neck pain.

## 1. Introduction

Non-specific neck pain (NNP) is a symptom related to postural or mechanical cause [1,2]. In most patients, neck pain can be a common cause of disability: it is associated with daily activity limitations, reduction of work productivity and decrease in quality of life [3,4]. Neck pain has a socioeconomic impact [5,6,7,8,9] because of physical and psychological symptoms that are related [10,11,12]. NNP’s prevalence increases in industrialized countries and in urban areas; it is generally higher in females and in middle age. About 80% of the general adult population suffers neck pain during their lifetimes, and 30–50% have neck pain annually [3,13]. Therefore, NNP is a symptom with a multifactorial etiology, and studies show its strong correlation with depression, anxiety, headache, sedentary life, sleep disorders and smoking [13,14,15]. Risk factors for developing NNP include cervical trauma such as whiplash, sports injuries and sedentary seated work [3,16,17]. Acute NNP lasts less than 4 weeks; subacute NPP has a duration of 1–4 months; and chronic neck pain persists more than 4 months [1]. Furthermore, neck pain can also be categorized by mechanism as mechanical when its origins are from the spine’s postural disorders, or neuropathic when it results in peripheral nervous system impairments with nerve roots’ irritation determined by herniated disk, osteophyte or spinal stenosis [18,19]. Diagnosis of NNP is based on clinical grounds in the absence of red flags suggesting more serious pathology (non-conventional musculoskeletal disorders such as spinal cord injury or non-musculoskeletal disease) [20]. The patient’s history and physical examination are, consequently, the first step to distinguish NP type [21]. Then, a plain radiograph of the cervical spine could help the diagnosis. An X-ray usually reveals loss of normal cervical lordosis, which explains cervical muscle spasm [22]. Soft tissue abnormalities could be examined with Magnetic Resonance Imaging (MRI), and computed tomography (CT) could help bone state analysis [23]. Finally, electromyography can complete the nerve conduction study when symptoms of nerve-root irritation occur [24]. Most NNP responds to conservative treatments, but the best therapeutic choice is still undefined. Physicians used to prescribe drugs such as NSAIDs, analgesics and muscle relaxants in combination with physiotherapy (manual therapy and exercise and/or instrumental physical therapies) [1]. In the rehabilitative field, Muscle Energy Technique (MET) is an active manual technique in which the physiotherapist does not control corrective force [25]. The patient, in fact, should be able to produce oriented voluntary contraction of varying intensity [26]. In 1989, Greenman PE described isometric, concentric and eccentric contractions as types of muscle contraction to perform in MET [27]. Therefore, it is a manual treatment to improve any articulation’s decreased range of motion. This technique can solve muscle contracture or weakness, and it can reduce localized oedema by stimulating rhythmic muscle movements [28]. MET decreases sympathetic tone through fascial stimulation and localized vasodilatation [29]. Then, the patient can perform an isometric contraction and, consequently, a post-isometric relaxation of the muscle contracted. MET also induces a reciprocal agonist muscle inhibition. This phenomenon is a result of a physiological neuro-response involving Golgi tendon organs. Moreover, a patient can produce movement performing an isotonic eccentric or concentric contraction when a therapist’s force overcomes or partially matches a patient’s effort respectively [30]. Therefore, MET is a ‘hands-on’ therapy to induce muscle stretching, strengthening and relaxing. It is a rehabilitative therapeutic option for non-specific neck pain with the aim to restore normal joint mobility and reduce pain [31]. As such, it would be desirable for the physiatrist and the physiotherapist to have available rehabilitative practice points in respect to specific MET protocols, perhaps also in association with conventional treatment for NNP. Thus, the aim of this systematic review was to investigate the effectiveness and safety of MET for non-specific acute and chronic neck pain. This paper is a summary for physicians to make updates regarding current evidence-based MET efficacy on NNP.

## 2. Methods

The Preferred Reporting Items for Systematic Reviews and Meta-Analyses (PRISMA) was used for this results review.

### 2.1. Eligibily Criteria

All clinical trials published from 2010 to January 2020, written in English and Italian and specifically dealing with the topic of the “muscle energy technique in aspecific neck pain” treatment, were included. Eligible studies reflected PICO criteria: (i) Population: Adult males and females with non-specific acute or chronic neck pain, caused by mechanical/mio-tensive impairments or other disorders. (ii) Interventions: Muscle Energy Technique as a rehabilitative choice for NNP. (iii) Comparisons: Patients received only traditional physiotherapy or other rehabilitative treatment (for example, stretching), or a combination of these therapies with muscle energy technique. (iv) Outcomes: Eligible studies needed to include at least one of the following outcomes: neck pain evaluated by clinical scales (Visual Analogic Scale-VAS, Numeric Pain Rating Scale-NPRS, Pressure-Pain Threshold-PPT); disability evaluated by NDI (Neck Disability Index) questionnaire; joint function evaluated by measurement of range of motion (ROM); quality of life, as evaluated by standard validated questionnaires. We excluded all studies without full-text available, written in other languages and without outcome measures, and articles about other interventions not involving MET.

Inclusion and exclusion criteria are summarized in Table 1.

### 2.2. Information Source and Searches

A literature search was conducted (December 2019–January 2020) using the following five databases: PubMed, Medline, PEDro, Cochrane Database, and Google Scholar. Keywords for investigation were identified using the contributing authors’ knowledge. Keywords used were “muscle energy technique AND/OR neck pain AND/OR upper trapezius trigger points, MET AND/OR neck pain AND/OR upper trapezius trigger points. Searches were supplemented by hand searching and retrieval of any additional articles meeting eligibility criteria that were cited in reference lists.

### 2.3. Study Selection

All records identified in the search were imported into Microsoft Excel. Two independent reviewers searched databases by using the same strategy to ensure proper cross-checking of the results. The authors evaluated the studies identified by the searches based on the inclusion and exclusion criteria established. The authors independently screened the titles, abstracts and full text of eligible studies. Studies marked as not meeting eligibility criteria were reviewed by two reviewers (C.L.R and S.S.) for confirmation. The result was a final set of full-text articles for data extraction and inclusion for qualitative analysis.

### 2.4. Data Collection

Data extracted from included studies comprised: authors and date of study; type of intervention, core components and diagnostic population; who delivered the intervention; intensity of the intervention; study design and original authors’ conclusions about efficacy across study outcomes. Where multiple studies existed, reviewers noted when the older research was superseded by newer evidence. All data required to answer the study questions were published within the papers; no contact with authors was necessary. Any disagreement regarding accepting full-text articles was resolved by discussion until consensus was reached.

### 2.5. Quality Assesment

Two authors (C.L.R. and S.S.) did the assessment of risk of bias independently. Risk of bias of controlled studies was assessed according to the Cochrane Collaboration’s domain-based evaluation framework [32]. Main domains were assessed in the following sequence: (1) selection bias (randomized sequence generation and allocation concealment); (2) performance bias (blinding of participants and personnel); (3) detection bias (blinding of outcome assessment); (4) attrition bias (incomplete outcome data, e.g., due to dropouts); (5) reporting bias (selective reporting); (6) other sources of bias. The scores for each bias domain and the final score of risk of systematic bias were graded as low, high or unclear risk. The methodological quality was also assessed using the Physiotherapy Evidence Database PEDro scale [33].

## 3. Results

### 3.1. Study Selections

**Figure 1:**
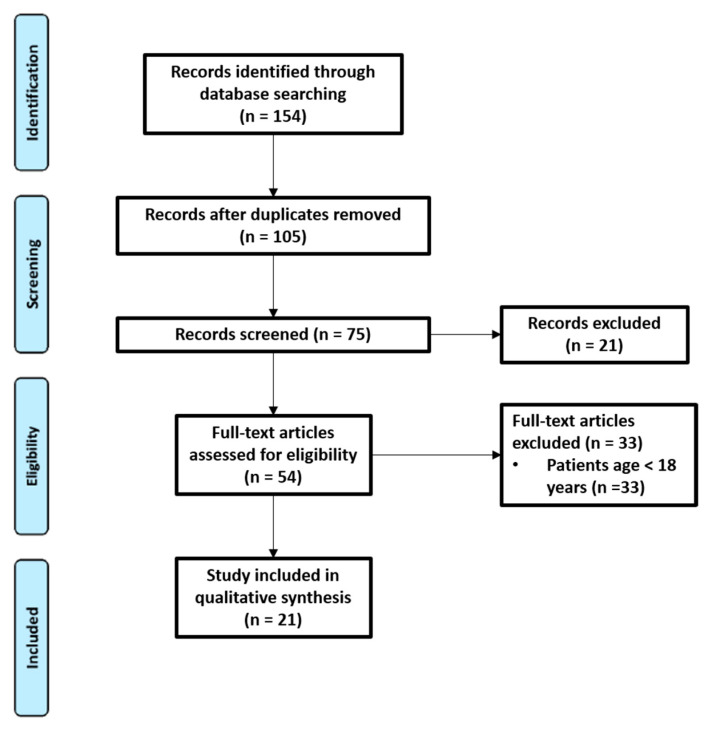
Flow chart.

As shown in the study flow chart (Figure 1), the literature search identified 154 records. After removing duplicates, the research resulted in 105 records. A total of 75 records were screened on the basis of their titles and abstracts. Then, 54 were discarded following application of the inclusion and exclusion criteria. Finally, 21 were considered relevant for qualitative analysis.

### 3.2. Characteristics of Included Studies

Characteristics of included studies are summarized in Table 2 and Table 3. We used 15 studies [34,35,36,37,38,39,40,41,42,43,44,45,46,47,48] to analyze the effects of MET in patients with non-specific acute neck pain, and 6 studies [49,50,51,52,53,54] to analyze the effects of MET in patients with non-specific chronic neck pain. Articles had sample sizes ranging from 28 to 90, contributing to a total sample size of 913 participants, consisting of 527 females and 386 males with a mean baseline age of 18 to 55 years with an average of 32.18 (±7.59). In each clinical trial, MET’s role was evaluated. Regarding non-specific acute neck pain treatment: 4 clinical trials compared MET with another technique, 6 studies evaluated the efficacy of MET in combination with traditional physical therapy (TPT) compared to one of these therapies alone; 5 studies compared MET’s effectiveness in association with other therapies with MET or another rehabilitative therapy such as ischemic compression (IC) alone. Similarly, regarding non-specific chronic neck pain treatment: 3 studies made a comparison between MET and another physical therapy technique; 2 clinical trials compared the effect of MET in conjunction with traditional physical therapy (TPT) with TPT or MET; 1 study compared the effectiveness of MET plus Deep Neck Flexors exercises with deep neck flexors exercises alone. Twenty clinical trials assessed neck pain; VAS was most commonly reported. Fourteen studies recorded disability with an NDI questionnaire and 15 studies measured joint function with cervical extension, cervical lateral, and anterior flexion and cervical rotation. These outcomes were measured at baseline and 1 or 2 weeks later. Some studies evaluated outcomes before and immediately after treatment (pre–post).

#### 3.2.1. Studies Characteristics about People with Non-Specific Acute Neck Pain

Four studies evaluated MET’s effectiveness in improving established outcomes. Most of these studies supported MET’s role to reduce pain and disability and increase cervical range of motion (Table 2). Gilani et al. [35] published a clinical trial in 2018. They randomly divided 30 patients into two groups: Group 1 (*n* = 15) received ischemic compression (IC) in upper trapezius myofascial trigger points, and Group 2 (*n* = 15) received muscle energy technique (MET). According to this study, IC was more significantly effective (*p* ≤ 0.001) for reducing neck pain than MET. MET, in contrast, was more effective for improving range of motion (*p* ≤ 0.001). The clinical trial of Kirthika et al. [40] examined the effect of MET and IC on upper trapezius myofascial trigger points. They divided 30 subjects into Group A (MET) and Group B (IC). Authors found statistically significant results in both group for pain or cervical ROM. In conclusion, MET was superior in improving cervical lateral flexion. Sata [47] published in 2012 a study that included 52 patients with myofascial trigger points on upper fibers of trapezius. Patients were randomly divided in two groups: one group was treated with MET and the other with myofascial release therapy. Finding data about pain intensity (VAS), neck disability index (NDI) and pressure pain threshold showed myofascial release therapy as a more effective treatment. According to CONSORT guidelines for RCTs, Nagrale et al. [48] demonstrated MET’s efficacy in improving VAS, NDI and lateral cervical flexion range of motion (ROM). By the way, findings of this single blinded randomized controlled trial were in favor of the integrated neuromuscular inhibition technique (INIT) [55]. INIT is the combination of MET, ischemic compression, and Strain Counter-Strain (SCS), and it was more beneficial than MET in isolation.

Six studies were considered about the efficacy of MET in combination with traditional physical treatment (TPT) compared with only TPT or TPT with other rehabilitative techniques. In an RCT published in 2018, Kashyap et al. [34] compared the clinical effect of manual pressure release (MPR) with the muscle energy technique (MET) for neck pain. The study enrolled 45 female participants (mean age ± SD = 21.49 ± 3.66; age range = 18–30 years). The outcomes measures were: (i) neck pain as evaluated by VAS scale; (ii) functional disability as evaluated by Neck Disability Index Questionnaire; and (iii) range of neck rotation. Outcome measures were evaluated respectively, at baseline (day 0), day 1, day 5, day 10 and day 15 post-intervention. According to the authors, MPR and the MET were equally effective (*p*-value < 0.05) for reducing pain and muscle tenderness. Moreover, both treatments could improve neck disability and range of rotation in patients with non-specific neck pain. Tank et al. [37] carried out a clinical trial on the effectiveness of MET and Mulligan SNAGS (MS) on pain, functional disability, and active cervical ROM for individuals with mechanical neck pain. They randomly divided 40 subjects into two groups: Group 1 (*n* = 20) received MET plus conventional therapy, and Group 2 (*n* = 20) received MS plus conventional therapy. Data showed equal effectiveness for neck pain, disability, and cervical ROM. These two techniques in combination with conventional therapy could be a rehabilitative option for non-specific acute neck pain. In an RCT published in 2016, Phadke et al. [38] tried to highlight the best long-term treatment for neck pain. They randomly placed 45 males with Myofascial Trigger Points Pain into three groups: Group A (*n* = 15) received MET with Strain Counter-Strain, Group B (*n* = 15) received MET, and Control Group C (*n* = 15) received conventional treatment. The primary outcome was PPT as assessed by pressure threshold meter (PTM). The secondary outcomes were pain (VAS) and functional status of the patients (NDI). According to the authors, after 1-week post-intervention, MET in association with Strain Counter-Strain produced greater improvement in the pain pressure threshold, function status and pain intensity. Shah et al. [42] analyzed the effects of MET and IC on pain, cervical ROM (CROM) and the pressure-pain threshold (PPT) of the upper trapezius trigger point. The authors showed statistically significant results in outcomes’ improvement: MET brought more benefits on improving CROM than IC. Yadav et al. [43] published a clinical trial about the effectiveness of MET on neck pain. A total of 33 patients including 18 males and 15 females were selected and randomly allocated into three groups. DNF (Deep Neck Flexors) training and MET had good clinical effects compared to traditional care in reducing functional disabilities, pain and improving ROM. DNF induced more statistically significant improvement of outcomes than MET. Richa et al. [46] (2012) examined three patients’ groups with mechanical neck pain. The authors’ aim was to find MET’s effectiveness when compared with static stretching exercises. In each patient, a significant decrease of pain intensity was reported after treatment. The analysis similarly revealed a significant improvement of active cervical ROM in all three groups. Therapists, then, should know both techniques to successfully treat mechanical subacute neck pain.

Five clinical trials about the efficacy of MET in association with other rehabilitative treatments were selected. Basak et al. [36] evaluated the effect of Ischemic compression (IC) and Dry Needling (DN) in combination with MET. The randomly allocated 28 individuals with upper trapezius Myofascial Trigger Points (MTrPs) into two treatments groups: Group 1 received IC and MET for 3 sessions for 1 week, and Group 2 received DN and MET for 3 sessions for 1 week. The authors found statistical and significant results in both groups on neck pain, cervical ROM and Neck Disability (*p* value < 0.05) after 1 week of intervention. IC and DN were equally effective in combination with MET in the treatment of upper trapezius MTrPs. Iqbal et al. [39] established the best possible long-term effective treatment by using the combination of muscle energy technique with the Strain Counter-Strain technique. They selected 45 individuals with upper trapezius MTrPs. The combination of two manual techniques produced greater improvement in pain pressure threshold, function status and reduction in pain intensity after 1 week of the end of treatment (*p* value ≤ 0.001). In a clinical trial, Yeganeh Lari et al. [41] investigated the combination of dry needling (DN) and MET on the upper trapezius latent myofascial trigger point. They randomly divided 60 female patients into three groups: Group 1 (*n* = 20) received DN and MET, Group 2 (*n* = 20) received only MET, and Group 3 (*n* = 20) received just DN. All groups showed decreases in pain (*p* 1⁄4 0.001) and an increase in pressure pain threshold levels (*p* 1⁄4 0.001) and in range of active contra lateral flexion (*p* 1⁄4 0.001). Moreover, a combination of DN and MET determined more significant improvement than single treatments. In a clinical trial published in 2015, Kumar Yatheendra [44] et al. compared the clinical effects of MET, IC and Strain Counter-Strain on upper trapezius trigger points in patients with mechanical neck pain. Authors found strong evidence for reducing pain and cervical function improving (*p* < 0.05) after the 4th week of each therapy. Moreover, MET resulted in superior treatment for upper trapezius trigger points. Nambi et al. [45] (2013) evaluated different effects of IC and MET on upper trapezius MTrPs. According to their clinical trial, MET significantly improves disability and cervical ROM (*p* value < 0.05). Regarding acute non-specific neck pain then, MET results show a beneficial treatment improving VAS, PPT, NDI and cervical spine function (ROM). By the way, patients reported improvements in acute neck pain and cervical movements when they experienced a combination of physical therapies including MET, as INIT for example. MET should have a key role in acute non-specific neck pain’s rehabilitative treatment to enhance conventional physical therapy techniques’ effect, making outcomes more long-lasting.

#### 3.2.2. Studies Characteristics about People with Non-Specific Chronic Neck Pain

Regarding non-specific chronic neck pain, a comparison between MET and other physical therapies was evaluated. Three clinical trials were considered. Jeong et al. [51] analyzed the impact of MET and passive stretching massage on ROM, strength, and pressure pain threshold (PPT) in patients with neck pain. They randomly assigned 30 subjects to three groups: Group 1 (*n* = 10) received passive stretching, Group 2 (*n* = 10) received massage, and Group 3 (*n* = 10) received MET. According to the authors, the proposed interventions were effective in improving outcome measures (*p* < 0.05). Further high-quality studies are needed to complete MET’s comparison with other therapies. Sadria et al. [52] compared the effect of MET and active release techniques (ART) on upper trapezius MTrPs. They selected 64 participants (32 males and 32 females). Both manual treatments showed significant results for decreasing pain (*p* value < 0.01) and increasing active range of cervical lateral flexion (*p* < 0.001). Kumari et al. [53] evaluated MET and Proprioceptive neuromuscular facilitation (PNF) in subjects with chronic mechanical neck pain. They randomly allocated 45 individuals in three treatment groups: Group A received 12 sessions of MET, Group B received 12 sessions of PNF technique, and Group C received 12 sessions of isometric and self-stretching exercise for 4 weeks. The outcome parameters (pain, ROM and function) were evaluated at baseline and after the treatment period. Authors found statistically significant improvement of outcomes (*p* value < 0.05) in both groups. These two methods brought benefits on decreasing pain, increasing ROM and improving function in chronic mechanical neck pain. The second topic is related to the efficacy of MET in association with traditional physical treatment (TPT) compared with only TPT or TPT with other available techniques. Two studies were evaluated. In a recent clinical trial, Zibiri et al. [49] compared the efficacy of muscle energy technique (MET) and neck stabilization exercise (NSE) on pain, neck disability, depression, anxiety, and sleep disturbance in patients with non-specific chronic neck pain (NSCNP). They divided 35 individuals into 3 groups: Group 1 (*n* = 12) received MET, neck care education (NCE), and infrared radiation (IR); Group 2 (*n* = 12) had NSE, neck care education (NCE), and IR; and Group 3 (*n* = 11) received NCE and IR. All patients had significant improvement in pain, disability, depression, anxiety and sleep disturbance. Notably, subjects treated with NSE showed more improvement in outcomes than others. El-Laithy et al. [50] investigated the effect of MET as post-isometric relaxation (PIR) in combination with the traditional physical therapy versus the traditional physical therapy alone. They randomly assigned 30 patients into two groups: Group 1 performed a traditional physical therapy program, and Group 2 received PIR plus a traditional physical therapy program. The authors found statistically significant results in reducing pain (*p* < 0.05) and ROM improvement (*p* value < 0.05) for both groups. Moreover, data showed strong evidence in favor of PIR combined with traditional physical therapy (*p* value < 0.05) in the neck pain treatment. Finally, one study evaluated the efficacy of Met in addition with another therapy in non-specific chronic neck pain’s treatment. Sakshi at al. [54] carried out a clinical trial on the effectiveness of MET combined with deep neck flexors (DNF) exercise in reducing pain, disability and correcting forward head posture in patients with chronic neck pain. A group of enrolled patients received MET combined with deep neck flexors exercises; another group received deep neck flexors exercises alone. The outcomes were recorded at baseline, day 14 and day 28. Data showed more evidence in favor of MET combined with deep neck flexors exercise than only deep neck flexors exercise in improving outcomes. MET, then, is an available good choice to treat non-specific chronic neck pain, as well as passive stretching, the active release technique, and proprioceptive neuromuscular facilitation. NSE in association with neck care education and infrared radiation could have more effect on reducing pain and disability.

### 3.3. Quality Assessment

The methodological quality of the 21 included articles, according to the PEDro scale, with averages between 3/10 to 9/10, averaged 6.91/10 (see Table 4). The risk of bias was considered low for 5 studies and high for 17 studies (see Table 5). The most frequent source of potential bias was the performance bias related to inadequate selected reported and incomplete outcome data.

## 4. Discussion

The aim of this review is to summarize the available evidence about MET efficacy in acute or chronic non-specific neck pain. Included RCTs have a “high–moderate” quality level as shown by quality assessment, even if analyzed studies achieved the lowest scores on item 6 (blinding of therapist) and item 11 (point and variability measures). The included studies analyzed many patients aged between 18 and 55 with a heterogeneity in terms of sex. Regarding the outcomes examined, such as pain, disability and joint function, the data that emerged are very homogeneous, and there is no significant difference in any of the studies analyzed. The first question was: “How effective is MET compared to other available therapies in non-specific acute or chronic neck pain?” For convenience, the studies were analyzed by dividing them into two subgroups, one treating acute non-specific neck pain and the other chronic neck pain, respectively. All the studies analyzed show an effective improvement in the outcomes of pain, disability and joint function, which leads us to say that MET is certainly an effective and safe technique in the treatment of cervical pain [35,40,47,48,49,50,51,52,53,54,55]. However, the results for the two subgroups were different: as regards neck pain in the acute phase, a significant improvement was recorded in all three outcomes, superior to or comparable to the other methods with which it was compared [35,40,47,48,55]; in the chronic phase neck pain there was greater attention in relation to joint function, the only parameter significantly higher than the other comparison methods [49,50,51,52,53,54]. The articles included are not enough to be able to say with certainty that MET is superior to other methods, and this is also because the comparison differs based on the administration of the techniques for the number of sessions and their duration. What emerges from all the studies in any case is the benefit that MET shows in the treatment of neck pain in both acute and chronic phases. Similarly for the second question: “Is MET combined with conventional therapy effective in the treatment of pain, disability and restoration of joint function in acute and chronic non-specific neck pain compared to conventional therapy alone or to the latter combined with another method?”, with regard to patients with neck pain in the acute phase and in the chronic phase, MET is very effective in improving health outcomes such as pain, disability and joint function. The results showed that the combination of conventional therapy, whether it is analgesic instrumental therapy, active stretching or postural education, and MET is very effective with significantly relevant results, in accordance with the guidelines for the treatment of cervical pain that recommend a multifactorial approach in the management of problems related to this pathology [34,37,38,42,43,46]. On this assumption, the third question was examined: “Can MET combined with other methods be effective in the treatment of acute and chronic nonspecific neck pain?”. The studies in this area were not sufficient to be able to state with certainty that the combination of MET and another method is effective, but in any case, in the included studies there is always a benefit in the combination of two methods, thus making MET a sometimes an added value in the treatment of cervical pain [36,39,41,45]. In conclusion, MET appears to be effective in the treatment of neck pain in both the acute and chronic phases. The greatest results were obtained by combining the MET method with conventional therapy—most often consisting of the use of instrumental physical therapy such as TENS, ultrasounds, or thermotherapy to relieve pain symptoms and the suggestion for the patient to perform a muscle stretching of the cervical tract, as well as strengthening exercises of the same muscles and the assumption of correct postures during the day in order to prevent the painful manifestations of the pathology sometimes caused by bad postural habits. The restoration of joint function is particularly significant, which consequently leads to reduced disability in patients with cervical pain, in particular in subjects with chronic neck pain. To obtain greater results and make the most of the potential of the method, it is necessary to combine it with other treatments, in particular conventional therapy, and to carry out the treatment for a period not limited to one session, as it has been seen that more sessions of MET lead to more stable results, sometimes preventing, due to the nature of the pathology and its progression, chronicization.

Further studies, therefore, are needed to clarify MET’s role, especially at long-term follow up. Overall, the analyzed clinical trials described several timings for MET training (1 session/week or 4 sessions/week). Data suggest that 2–3 sessions per week are needed to reach complete pain resolution. From our analysis, a combined approach (MET plus tradition physical therapy or stretching or neck stabilization exercises) could be a good therapeutic choice in chronic and acute non-specific neck pain management. However, the cause of reported improvement remains unclear after techniques’ combination training. MET improves the musculoskeletal system’s function (ROM), and it reduces acute and chronic neck pain. MET also requires patients’ active involvement during the treatment, through isometric and/or isotonic contractions.

To our knowledge, this is the first available review on MET for people with non-specific neck pain. The strengths of this review include the results’ applicability and systematic research methodology. Each step involved three independent authors, limiting potential errors. The limitations of this review are non-RCT designs and the low methodological quality of the included studies that limits the interpretability of the results.

## 5. Conclusions

The present study provides evidence that the use of MET in acute or chronic non-specific neck pain could be a good therapeutic rehabilitative choice. Nonetheless, further studies are needed to define a standard treatment time, especially with respect to the proposal of overlapping MET protocols and on the timing of the start of rehabilitative therapy in the presence of acute cervical pain. The results suggest that MET therapy is more effective when performed in combination with other conventional rehabilitation therapies. Overall, there is a lack of high-quality evidence investigating the effectiveness and safety of MET to guide its use in the clinical management of non-specific neck pain. However, the high risk of bias and methodological shortcomings require caution in interpreting these results.

## Figures and Tables

**Table 1:** Inclusion and exclusion criteria used for paper selection.

Inclusion Criteria	Exclusion Criteria
(i) All clinical trials	(i) Narrative review/systematic review/meta-analysis
(ii)Published between 2010 and January 2020	(ii) Published before 2010
(iii) English language	(iii) Other languages
(iv) Full text available	(iv) Full text not available
(v) Patients with non-specific acute or chronic neck pain, caused by mechanical/mio-tensive impairments or other disorders	(v) Populations with other diseases
(vi) Male and female	(vi) Aged <18 years old
(vii) Aged >18 years old	(vii) Rehabilitation program not including MET
(viii) Rehabilitation program including MET	(viii) All studies not including aforementioned outcomes
(ix) All studies including at least one of following outcomes (neck pain, disability joint function and quality of life).	

**Table 2:** Description of intervention in non-specific acute neck pain.

	Study Population	Method	Dosages and Frequency of Intervention	Assessment Intervals	Outcome Measures	Conclusions
Kashyap et al. (2018) [34]	Group 1 (*n* = 15) 21.27 ± 3.86 years Group 2 (*n* = 15) 22.07 ± 4.11 years Group 3 (*n* = 15) 21.13 ± 3.00 years	TPT+ MPR TPT+ MET TPT	Four times a lesson	T1: baseline T2: 5 days T3: 10 days T4: 15 days	Neck Pain: (VAS-PPT) Disability (NDI) Joint function (ROM)	MPR and MET are equally effective for decreasing pain intensity and functional disability of the neck (*p* value < 0.05)
Gilani et al. (2018) [35]	Group 1 (*n* = 15) Group 2 (*n* = 15)	MET IC	12 series/lessons for 4 weeks	T1: baseline T2: 1-week T3: 2 weeks T4: 3 weeks T5: 4 weeks	Neck Pain: (VAS-NPRS) Disability (NDI) Joint function (ROM)	IC and MET was more effective for improving range of motion and for reducing neck pain (*p* value = 0.000).
Basak et al. (2018) [36]	Group 1 (*n* = 14) Group 2 (*n* = 14)	IC and MET DN and MET	Three times a week	T1: baseline T2: 1 week	Neck Pain: (PPA-NPDA) Joint function (STG)	IC and DN were equally effective in combination with MET in the treatment of upper trapezius MTrPs. (*p* value < 0.05)
Tank et al. (2018) [37]	Group 1 (*n* = 20) Group 2 (*n* = 20)	MET and TPT MS and TPT	MET: Three times a week MS: Three times a week TPT: Six times a week	T1: baseline T2: 2 weeks	Neck Pain: (VAS) Disability (NDI) Joint function (ROM)	MET and MS plus conventional therapy could be used as alternative treatment for nonspecific acute neck pain.
Phadke et al. (2016) [38]	Group 1 (*n* = 28) Group 2 (*n* = 28)	MET and TPT MS and TPT	Six times a week	T1: baseline T2: 6 days	Neck Pain: (VAS) Disability (NDI)	MET with strain-counter strain produced greater improvement in pain pressure threshold, function status and reduced pain intensity.
Iqbal et al. (2016) [39]	Group 1 (*n* = 15) Group 2 (*n* = 15) Group 3 (*n* = 15)	MET and strain MET TPT		T1: baseline T2: 1-day T3: 5 days	Neck Pain: (VAS-PPT) Disability (NDI)	The combination of MET and strain technique produced improvement in pain pressure threshold, function status and reduction in pain (*p* value < 0.000).
Kirthika et al. (2016) [40]	Group 1 (*n* = 15) Group 2 (*n* = 15)	MET IC		T1: pre-test T2: post test	Neck Pain: (VAS) Joint function (ROM)	MET was superior to IC in improving cervical lateral flexion.
Yehaneh Lari et al. (2015) [41]	Group 1 (*n* = 20) 25.60 ± 0.8 years Group 2 (*n* = 20) 24.78 ± 0.7 years Group 3 (*n* = 20) 24.60 ± 0.9 years	DN and MET MET DN	3 lessons MET: 3/5 repetitions a lesson	T1: baseline T2: second lessons T3: third lessons T4: follow up	Neck Pain: (VAS-PPT) Joint function (ROM)	Group 1 showed more significant improvement than the other two groups. (*p* value < 0.001)
Shah et al. (2015) [42]	Group 1 (*n* = 15) 33.2 ± 3.61 years Group 2 (*n* = 15) 35.66 ± 5.32 years	MET and TPT IC and TPT	One week	T1: baseline T2: 6 days	Neck Pain: (VAS-PPT) Joint function (ROM)	MET brought more benefits on improving ROM than IC.
Yadav et al. (2015) [43]	Group 1 (*n* = 30) Group 2 (*n* = 30) Group 3 (*n* = 30) Age between 18 to 45 years	TPT DNF and TPT MET and TPT	Five times a week for two weeks	T1: baseline T2: 1-week T3: 2 weeks	Neck Pain: (VAS) Joint function (ROM) Disability (NDI)	MET had statistically more significant improvement of outcomes.
Yatheendra Kumar et al. (2015) [44]	Group 1 (*n* = 30) Group 2 (*n* = 30) Group 3 (*n* = 30)	MET and TENS IC and TENS SCS and TENS	Three times a week for four weeks	T1: baseline T2: 2 weeks T3: 4 weeks	Neck Pain: (VAS) Joint function (ROM) Disability (NDI)	MET was superior in the treatment of upper trapezius trigger points.
Nambi et al. (2013) [45]	Group 1 (*n* = 15) 46.20 ± 5.88 years Group 2 (*n* = 15) 45.46 ± 5.44 years	MET and US IC and US	Three times a week for four weeks	T1: baseline T2: 4 weeks	Neck Pain: (VAS) Joint function (ROM)	MET significantly improves disability and cervical ROM. (*p* value < 0.05)
Richa et al. (2012) [46]	Group 1 (*n* = 15) Group 2 (*n* = 15) Group 3 (*n* = 15) 18–43 years	MET and TPT STRETCHING and TPT TPT	For two weeks MET: 6 sessions TPT: 10 sessions Stretching: 6 sessions	T1: baseline T2: 2 weeks	Neck Pain: (VAS) Joint function (ROM) Disability (NDI)	MET and stretching techniques treated successfully mechanical subacute neck pain
Sata et al. (2012) [47]	Group 1 (*n* = 25) 30.80 ± 5.36 years Group 2 (*n* = 27) 29.44 ± 5.38 years	MET MRT	Six times a week	T1: baseline T2: 1 week	Neck Pain: (VAS-PPT) Disability (NDI)	Met was more effective treatment
Nagrale et al. (2010) [48]	Group 1 (*n* = 32) Group 2 (*n* = 32) 19–38 years	MET INIT	Four weeks	T1: baseline T2: 2 weeks T3:4 weeks	Neck Pain: (VAS) Joint function (ROM) Disability (NDI)	INIT resulted more beneficial than MET in isolation

Legend: DN: dry needling; DNF: deep neck flexors; IC: ischemic compression; INIT: integrated neuromuscular inhibition technique; MET: muscle energy technique; MPR: manual pressure release; MRT: myofascial release therapy; MS: mulligan snags; NDI: neck disability index; NPAD: neck pain and disability scale; NPRS: numerical pain rating scale; PPA: pain pressure algometer; PPT: pressure pain threshold; ROM: range of motion; STG: spin T goniometer; TPT: traditional physiotherapy treatment; US: ultrasound; VAS: visual analogue scale.

**Table 3:** Description of intervention in non-specific chronic neck pain.

	Study Population	Method	Dosages and Frequency of Intervention	Assessment Intervals	Outcome Measure	Conclusions
Zibiri et al. (2019) [49]	Group 1 (*n* = 12) 49.50 ± 17.5 years Group 2 (*n* = 12) 42 ± 14.58 years Group 3 (*n* = 11) 49.27 ± 11.32 years	MET, NCE and IR NSE, NCE and IR, NCE and IRR	Two times a week for 8 weeks.	T1: baseline T2: 4 weeks T3: 8 weeks	Disability (NDI) Pain (NPRS) Quality of life (HADS and ISI)	NSE provides a better benefit than MET and NCE. *p* value = 0.002
El Laithy et al. (2018) [50]	Group 1 (*n* = 15) 34.86 ± 8.39 years Group 2 (*n* = 15) 32.46 ± 6.54 years	MET and TPT TPT	-	T1: baseline T2: post session	Disability (NDI) Joint function (ROM) Pain (NRS)	MET was more effective in reducing pain and functional disability and increasing cervical ROM than the traditional treatment program alone. *p* value < 0.05
Jeong et al. (2017) [51]	Group 1 (*n* = 10) Group 2 (*n* = 10) Group 3 (*n* = 10) 21.5 ± 1.52 years	Stretching Massage MET	-	T1: baseline T2: post session	Pain (PPT) Joint function (ROM and muscle strength)	MET was more effective in reducing pain and increasing cervical ROM and muscle strength than the other treatment. *p* value < 0.05
Sadria et al. (2016) [52]	Group 1 (*n* = 32) 27.06 ± 8.54 years Group 2 (*n* = 32) 28.19 ± 9.77 years	ART MET	15 min	T1: baseline T2: post 2 h after session	Pain (PPT) Joint function (ROM)	MET and ART techniques can increase cervical ROM and reduce pain. *p* value < 0.01
Kumari et al. (2016) [53]	Group 1 (*n* = 15) 31.53 ± 10.06 years Group 2 (*n* = 15) 35.53 ± 8.39 years Group 3 (*n* = 15) 34 ± 8.77 years	MET PNF Isometric and self-stretching exercise	12 series/lessons for 4 weeks 12 series/lessons for 4 weeks 4 weeks	T1: baseline T2: post session	Pain (PPT) Joint function (ROM) Disability (NDI)	MET and PNF are both equally effective in reducing pain, improving ROM and function. *p* value < 0.01 There was no statistically significant difference between group 1 and group 2 *p* value = 0.88
Sakshi at al. (2014) [54]	Group 1 (*n* = 15) Group 2 (*n* = 15)	MET and DNF DNF	Over 4 weeks	T1: baseline T2: 2 weeks T3: 4 weeks	Disability (NDI) Pain (NRS)	MET combined with DNF exercises is more effective intervention for reducing pain and disability. *p* value < 0.01

Legend: ART: active release technique; DNF: deep neck flexors; HADS: hospital anxiety depression scale; IR: infra-red-radiation; IRR: infra-red radiation; ISI: insomnia severity index; MET: muscle energy technique; NCE: neck care education NDI: neck disability index; NPRS: numerical pain rating scale; NSE: neck stabilization exercise; PNF: proprioceptive neuromuscular facilitation; PPT: pressure pain threshold; ROM: range of motion; TPT: traditional physiotherapy treatment.

**Table 4:** Pedro score scale.

	Eligibility Criteria	Random Allocation	Allocation Concealed	Similar at Baseline	Subject Blinded	Therapist Blinded	Assessor Blinded	Outcome Measure	Intent to Treat	Between-Group Comparison	Point and Variability Measures	Score
Kashyap (2018) [34]	+	+	−	+	−	−	−	+	+	+	−	5/10
Gilani (2018) [35]	+	+	+	+	+	−	+	+	+	+	+	9/10
Basak (2018) [36]	−	+	+	−	−	−	−	+	+	+	−	5/10
Tank (2018) [37]	+	+	+	+	+	−	+	+	+	+	−	8/10
Phadke (2016) [38]	+	+	+	−	+	−	+	+	+	−	−	6/10
Iqbal (2016) [39]	+	−	−	−	+	−	+	+	+	−	−	4/10
Kirthika (2016) [40]	−	+	+	−	−	−	−	+	+	−	−	3/10
Yehaneh Lari (2015) [41]	−	+	+	+	−	−	−	+	+	+	−	6/10
Shah (2015) [42]	−	+	+	+	−	−	−	+	+	+	−	6/10
Yadav (2015) [43]	+	+	+	−	−	−	−	+	+	+	−	5/10
Yatheendra (2015) [44]	−	+	+	+	−	−	−	+	+	+	−	6/10
Nambi (2013) [45]	+	−	−	+	−	−	−	−	+	+	−	3/10
Richa (2012) [46]	−	+	+	+	−	−	−	+	+	+	−	6/10
Sata (2012) [47]	+	+	+	+	−	−	−	+	+	+	−	6/10
Nagrale (2010) [48]	+	+	+	+	+	−	−	+	+	+	+	8/10
Zibiri (2019) [49]	+	+	+	+	+	−	+	+	+	+	−	8/10
El-Laithy (2018) [50]	+	+	+	+	+	−	+	+	+	+	−	8/10
Jeong (2017) [51]	−	+	+	+	+	−	−	+	+	+	−	7/10
Sadria (2016) [52]	+	+	+	+	+	−	+	+	+	+	−	8/10
Kumari (2016) [53]	+	+	+	+	+	−	+	+	+	+	−	8/10
Sakshi (2014) [54]	+	−	−	+	−	−	−	+	+	+	−	4/10

Legend: “+” yes; “−“ no.

**Table 5:** Risk of bias.

	Random Sequence Generation	Allocation Concealment	Blinding of Partecipants	Blinding of Therapist	Blinding of Outcome Assessment	Incomplete Outcome Data	Selective Reporting	Rate
Kashyap (2018) [34]	+	+	−	−	−	+	+	Hight
Gilani (2018) [35]	+	+	?	−	−	+	+	Hight
Basak (2018) [36]	?	?	−	−	−	+	+	Hight
Tank (2018) [37]	?	?	?	−	−	+	+	Hight
Phadke (2016) [38]	+	+	?	−	−	+	+	Hight
Iqbal (2016) [39]	?	?	?	−	−	+	+	Hight
Kirthika (2016) [40]	?	?	?	−	−	?	?	Low
Yehaneh Lari (2015) [41]	?	?	−	−	−	+	+	Low
Shah (2015) [42]	?	?	?	−	−	+	+	Hight
Yadav (2015) [43]	+	+	?	−	−	+	+	Hight
Yatheendra Kumar (2015) [44]	?	?	?	−	−	+	+	Hight
Nambi (2013) [45]	−	−	?	−	−	+	+	Low
Richa (2012) [46]	?	?	−	−	−	+	+	Low
Sata (2012) [47]	?	?	?	−	−	+	+	Hight
Nagrale (2010) [48]	+	+	?	+	?	+	+	Hight
Zibiri (2019) [49]	+	+	?	−	−	+	+	Hight
El-Laithy (2018) [50]	+	+	?	−	−	+	+	Hight
Jeong (2017) [51]	?	?	?	−	−	+	+	Hight
Sadria (2016) [52]	+	+	+	−	−	+	+	Hight
Kumari (2016) [53]	?	?	?	−	−	+	+	Hight
Sakshi (2014) [54]	−	−	?	−	−	+	+	Low

Legend: “+”: Low Risk; “−“: High Risk; “?”: Unclear Risk.

## Data Availability

In this study no data was reported.

## References

[1] Binder A. (2007). The diagnosis and treatment of nonspecific neck pain and whiplash. Eur. Medicophys..

[2] Mangone M., Paoloni M., Procopio S., Venditto T., Zucchi B., Santilli V., Paolucci T., Agostini F., Bernetti A. (2020). Sagittal spinal alignment in patients with ankylosing spondylitis by rasterstereographic back shape analysis: An observational retrospective study. Eur. J. Phys. Rehabil. Med..

[3] Côté P., Cassidy J.D., Carroll L. (1998). The Saskatchewan Health and Back Pain Survey. The prevalence of neck pain and related disability in Saskatchewan adults. Spine.

[4] Bernetti A., Agostini F., de Sire A., Mangone M., Tognolo L., Di Cesare A., Ruiu P., Paolucci T., Invernizzi M., Paoloni M. (2021). Neuropathic Pain and Rehabilitation: A Systematic Review of International Guidelines. Diagnostics.

[5] Murray C.J., Atkinson C., Bhalla K., Birbeck G., Burstein R., Chou D., Dellavalle R., Danaei G., Ezzati M., Fahimi A. (2013). U.S. Burden of Disease Collaborators. The state of US health, 1990–2010: Burden of diseases, injuries, and risk factors. JAMA.

[6] Masiero S., Litwocenko S., Agostini F. (2020). On behalf section of Rehabilitation in Environmental Thermal for Italian Society of Physical Medicine and Rehabilitation. Rehabilitation in an Italian thermal setting: A new therapeutic strategy for patients with musculoskeletal disability-the results of an Italian survey. Int. J. Biometeorol..

[7] Damiani C., Mangone M., Paoloni M., Goffredo M., Franceschini M., Servidio M., Pournajaf S., Santilli V., Agostini F., Bernetti A. (2020). Trade-Offs with rehabilitation Effectiveness (REs) and Efficiency (REy) in a sample of Italian disabled persons in a in post-acuity rehabilitation unit. Ann. Ig..

[8] Masiero S., Maccarone M.C., Agostini F. (2020). Health resort medicine can be a suitable setting to recover disabilities in patients tested negative for COVID-19 discharged from hospital? A challenge for the future. Int. J. Biometeorol..

[9] Seccia R., Boresta M., Fusco F., Tronci E., Di Gemma E., Palagi L., Mangone M., Agostini F., Bernetti A., Santilli V. (2020). Data of patients undergoing rehabilitation programs. Data Brief..

[10] Fejer R., Kyvik K.O., Hartvigsen J. (2006). The prevalence of neck pain in the world population: A systematic critical review of the literature. Eur. Spine J..

[11] Borghouts J.A.J., Koes B.W., Vondeling H., Bouter L.M. (1999). Cost-of-illness of neck pain in The Netherlands in 1996. Pain.

[12] Hincapié C.A., Cassidy J.D., Côté P., Carroll L.J., Guzmán J. (2010). Whiplash injury is more than neck pain: A population-based study of pain localization after traffic injury. J. Occup. Environ. Med..

[13] Paolucci T., Cardarola A., Colonnelli P., Ferracuti G., Gonnella R., Murgia M., Santilli V., Paoloni M., Bernetti A., Agostini F. (2020). Give me a kiss! An integrative rehabilitative training program with motor imagery and mirror therapy for recovery of facial palsy. Eur. J. Phys. Rehabil. Med..

[14] Strine T.W., Hootman J.M. (2007). US national prevalence and correlates of low back and neck pain among adults. Arthritis Rheum..

[15] Son K.M., Cho N.H., Lim S.H., Kim H.A. (2013). Prevalence and risk factor of neck pain in elderly Korean community residents. J. Korean Med. Sci..

[16] Paolucci T., Agostini F., Mangone M., Bernetti A., Cordiani B., Bellomo R.G., Saggini R., Villani C. (2021). Sagittal spine alignment and postural balance in pre-puberty age: A multidisciplinary and multi-professional rehabilitative point of view. J. Biol. Regul. Homeost. Agents.

[17] Mangone M., Bernetti A., Agostini F., Paoloni M., De Cicco F.A., Capobianco S.V., Bai A.V., Bonifacino A., Santilli V., Paolucci T. (2019). Changes in Spine Alignment and Postural Balance After Breast Cancer Surgery: A Rehabilitative Point of View. Biores. Open Access..

[18] Cohen S.P. (2015). Epidemiology, diagnosis, and treatment of neck pain. Mayo Clin. Proc..

[19] de Sire A., Agostini F., Lippi L., Mangone M., Marchese S., Cisari C., Bernetti A., Invernizzi M. (2021). Oxygen-Ozone Therapy in the Rehabilitation Field: State of the Art on Mechanisms of Action, Safety and Effectiveness in Patients with Musculoskeletal Disorders. Biomolecules.

[20] Downie A., Williams C.M., Henschke N., Hancock M.J., Ostelo R.W., de Vet H.C., Macaskill P., Irwig L., van Tulder M.W., Koes B.W. (2013). Red flags to screen for malignancy and fracture in patients with low back pain: Systematic review. BMJ.

[21] Cohen S.P., Hooten W.M. (2017). Advances in the diagnosis and management of neck pain. BMJ.

[22] Gore D.R., Sepic S.B., Gardner G.M. (1986). Roentgenographic findings of the cervical spine in asymptomatic people. Spine.

[23] Bono C.M., Ghiselli G., Gilbert T.J., Kreiner D.S., Reitman C., Summers J.T., Baisden J.L., Easa J., Fernand R., North American Spine Society (2011). An evidence-based clinical guideline for the diagnosis and treatment of cervical radiculopathy from degenerative disorders. Spine J..

[24] American Association of Electrodiagnostic Medicine, So YT (1999). Guidelines in electrodiagnostic medicine. Practice parameter for needle electromyographic evaluation of patients with suspected cervical radiculopathy. Muscle Nerve Suppl..

[25] Goodridge J.P. (1981). Muscle energy technique: Definition, explanation, methods of procedure. J. Am. Osteopath. Assoc..

[26] Wilson E., Payton O., Donegan-Shoaf L., Dec K. (2003). Muscle energy technique in patients with acute low back pain: A pilot clinical trial. J. Orthop. Sports Phys. Ther..

[27] Greenman P.E. (1989). Principles of Manual Medicine.

[28] Lenehan K.L., Fryer G., McLaughlin P. (2003). The effect of muscle energy technique on gross trunk range of motion. J. Osteopath. Med..

[29] Schleip R. (2003). Fascial plasticity, a new neurobiological explanation—Part 2. J. Bodyw. Mov. Ther..

[30] Lewit K. (1999). Manipulative Therapy in Rehabilitation of the Motor System.

[31] Franke H., Fryer G., Ostelo R.W., Kamper S.J. (2015). Muscle energy technique for non-specific low-back pain. Cochrane Database Syst. Rev..

[32] Higgins J., Green S. (2011). Cochrane Handbook for Systematic Reviews of Interventions.

[33] de Morton N.A. (2009). The PEDro scale is a valid measure of the methodological quality of clinical trials: A demographic study. Aust. J. Physiother..

[34] Kashyap R., Iqbal A., Alghadir A.H. (2018). Controlled intervention to compare the efficacies of manual pressure release and the muscle energy technique for treating mechanical neck pain due to upper trapezius trigger points. J. Pain Res..

[35] Gilani M.H.Z., Obaid S., Tariq M. (2018). Comparison between effectiveness of ischemic compression and muscle energy technique in upper trapezius myofascial trigger points. Isra Med. J..

[36] Basak T., Pal T.K., Sasi M.M., Agarwal S. (2018). A Comparative Study on the Efficacy of Ischaemic Compression and Dry Needling with Muscle Energy Technique in Patients with Upper Trapezius Myofascial Trigger Points. Int. J. Health Sci. Res..

[37] Tank K.D., Choksi P., Makwana P. (2018). To study the effect of muscle energy technique versus mulligan snags on pain, range of motion and functional disability for individuals with mechanical neck pain: A comparative study. Int. J. Physiother. Res..

[38] Phadke A., Bedekar N., Shyam A., Sancheti P. (2016). Effect of muscle energy technique and static stretching on pain and functional disability in patients with mechanical neck pain: A randomized controlled trial. Hong Kong Physiother. J..

[39] Iqbal A., Ahmed H., Shaphe A. (2013). Efficacy of muscle energy technique in combination with strain-counterstrain technique on deactivation of trigger point pain. Indian J. Physiother. Occup. Ther..

[40] Kirthika V., Gopalakrishnan R., Gopinath Y., Revathy K., Thaslim K.F. (2016). A comparative study on the effectveness of muscle energy technique and ischaemic compression with ultrasound on upper trapezius myofascial trigger points. Int. J. Orthop. Surg. Implant. Technol..

[41] Yeganeh Lari A., Okhovatian F., Naimi S.S., Baghban A.A. (2016). The effect of the combination of dry needling and MET on latent trigger point upper trapezius in females. Man. Ther..

[42] Shah N.A., Shah N. (2015). Comparison of two treatment techniques: Muscle energy technique and Ischemic compression on upper trapezius trigger point in subjects with non- specific neck pain. Int. J. Ther. Rehabil..

[43] Yadav H., Goyal M. (2015). Efficacy of muscle energy technique and deep neck flexors training in mechanical neck pain- a randomized clinical trial. Int. J. Ther. Rehabil..

[44] Yatheendra Kumar G., Sneha P., Sivajyothi N. (2015). Effectiveness of Muscle energy technique, Ischaemic compression and Strain counterstrain on Upper Trapezius Trigger Points: A comparative study. Int. J. Phys. Educ. Sports Health.

[45] Nambi G.S., Sharma R., Inbasekaran D., Vaghesiya A., Bhatt U. (2013). Difference in effect between ischemic compression and muscle energy technique on upper trepezius myofascial trigger points: Comparative study. Int. J. Health Allied Sci..

[46] Richa M., Chitra K., Kshitija B. (2012). Comparative Effectiveness of Muscle Energy Technique and Static Stretching for Treatment of Subacute Mechanical Neck Pain. Int. J. Remote Sens..

[47] Sata J. (2012). A comparative study between muscle energy technique and myofascial release therapy on myofascial trigger points in upper fibres of trapezius. Indian J. Physiother. Occup. Ther..

[48] Nagrale A.V., Glynn P., Joshi A., Ramteke G. (2010). The efficacy of an integrated neuromuscular inhibition technique on upper trapezius trigger points in subjects with non-specific neck pain: A randomized controlled trial. J. Man. Manip. Ther..

[49] Zibiri R.A., Akodu A.K., Okafor U.A. (2019). Effects of muscle energy technique and neck stabilization exercises on pain, psychological status and sleep disturbance in patients with non-specific chronic neck pain. Middle East J. Rehabil. Health Stud..

[50] El-Laithy M.H., Fouda K.Z. (2018). Effect of post isometric relaxation technique in the treatment of mechanical neck pain. Phys. Ther. Rehabil..

[51] Jeong H.M., Shim J.H., Hye R.S. (2017). The passive stretching, massage, and muscle energy technique effects on range of motion, strength, and pressure pain threshold in musculoskeletal neck pain of young adults. Phys. Ther. Rehabil. Sci..

[52] Sadria G., Hosseini M., Rezasoltani A., Akbarzadeh Bagheban A., Davari A., Seifolahi A. (2017). A comparison of the effect of the active release and muscle energy techniques on the latent trigger points of the upper trapezius. J. Bodyw. Mov. Ther..

[53] Kumari C., Sarkar B., Banerjee D., Alam S., Sharma R., Biswas A. (2016). Efficacy of muscle energy technique as compared to proprioceptive neuromuscular facilitation technique in chronic mechanical neck pain: A randomized controlled trial. Int. J. Health Sci. Res..

[54] Sakshi N., Suman M., Geetanjali S. (2014). Effect of muscle energy technique and deep neck flexors exercise on pain, disability and forward head posture in patients with chronic neck pain. Indian J. Physiother. Occup. Ther..

[55] Chaitow L. (1994). INIT in treatment of pain and trigger points. Br. J. Osteopathy.

